# Crystal structure and Hirshfeld surface analysis of (succinato-κ*O*)[*N*,*N*,*N*′,*N*′-tetra­kis­(2-hy­droxy­eth­yl)ethyl­enedi­amine-κ^5^ *O*,*N*,*N*′,*O*′,*O*′′]nickel(II) tetra­hydrate

**DOI:** 10.1107/S2056989018015359

**Published:** 2018-11-06

**Authors:** Sevgi Kansiz, Necmi Dege, Yildiray Topcu, Yusuf Atalay, Snizhana V. Gaidai

**Affiliations:** a Ondokuz Mayıs University, Faculty of Arts and Sciences, Department of Physics, 55139, Kurupelit, Samsun, Turkey; b Ondokuz Mayıs University, Faculty of Engineering, Chemical Engineering Department, 55139, Samsun, Turkey; c Sakarya University, Faculty of Arts and Sciences, Department of Physics, 54187, Sakarya, Turkey; d Taras Shevchenko National University of Kyiv, Department of Chemistry, 64, Vladimirska Str., Kiev 01601, Ukraine

**Keywords:** crystal structure, nickel(II) complex, succinic acid, Hirshfeld surface

## Abstract

In the title compound, [Ni(C_10_H_24_N_2_O_4_)(C_4_H_4_O_4_)]·4H_2_O, the Ni^II^ cation is octa­hedrally coordinated by one O atom of the succinate anion and three O atoms and two N atoms from an *N*,*N*,*N*′,*N*′-tetra­kis­(2-hy­droxy­eth­yl)ethyl­enedi­amine mol­ecule. In the crystal, mol­ecules are linked by O—H⋯O and C—H⋯O hydrogen bonds, forming a three-dimensional supra­molecular architecture. Hirshfeld surface analyses and two-dimensional fingerprint plots were used to analyse the inter­molecular inter­actions present in the crystal, indicating that the most important contributions for the crystal packing are from H⋯H (63.3%) and H⋯O/O⋯H (34.5%) inter­actions.

## Abstract 2

One O atom of the succinate anion and three O atoms and two N atoms from a tetra­kis­(2-hy­droxy­eth­yl)ethyl­enedi­amine ligand coordinate to the Ni^II^ cation to form the complex which has a distorted octa­hedral geometry.

## Chemical context   

Aliphatic di­carb­oxy­lic acid ligands have been utilized consistently in the synthesis of a diverse range of metal complexes. The metal-ion geometries of coordination compounds can easily be identified. Transition metal atoms can be bridged by aliphatic or aromatic di­carboxyl­ate ligands to produce chains, layers and frameworks (Pavlishchuk *et al.*, 2011 ▸; Cheng *et al.*, 2013 ▸; Şen *et al.*, 2017 ▸). In addition, many transition and heavy metal cations play an important role in biological processes in the formation of many vitamins and drug components. An important element for biological systems is nickel. Nickel complexes have biological applications as a result of their anti­epileptic, anti­microbial, anti­bacterial and anti­cancer activities (Bombicz *et al.*, 2001 ▸). Nickel complexes with succinic acid [chemical formula (CH_2_)_2_(CO_2_H)_2_] are examples containing a di­carb­oxy­lic acid. The carboxyl O atoms ligate to transition metals and thus the succinic acid can bridge between nickel metal centres to form one-, two- and three-dimensional structures as polymeric chains, layers and frameworks, respectively. We describe herein the synthesis and structural features of a new Ni^II^ complex, namely (succinato-κ*O*)[*N*,*N*,*N*′,*N*′-tetra­kis­(2-hy­droxy­eth­yl)ethyl­ene­di­amine-κ^5^ *O*,*N*,*N*′,*O*′,*O*′′]nickel(II) tetra­hydrate. In addition, to understand the inter­molecular inter­actions in the crystal structure, Hirshfeld surface analysis was performed.

## Structural commentary   

**Figure 1:**
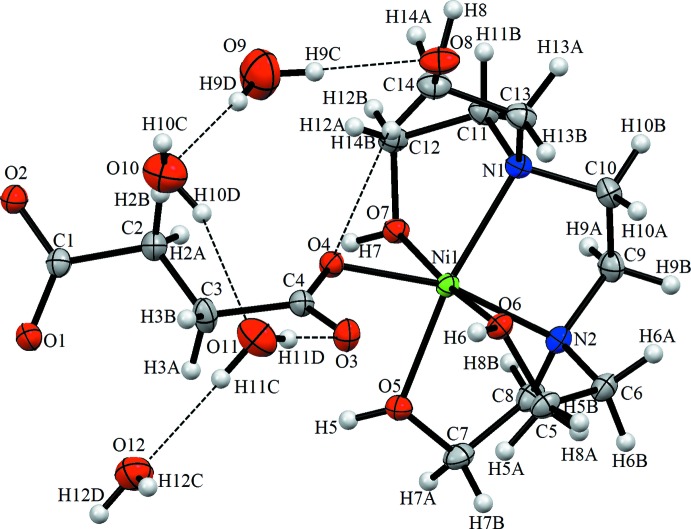
The mol­ecular structure of the title compound, showing the atom labelling. Displacement ellipsoids are drawn at the 20% probability level.

The mol­ecular structure of the asymmetric unit of the title compound is illustrated in Fig. 1 ▸. The Ni^II^ ion is octa­hedrally coordinated by three O atoms and two N atoms of *N*,*N*,*N*′,*N*′-tetra­kis­(2-hy­droxy­eth­yl)ethyl­enedi­amine mol­ecule and one O atom of the succinate anion. The Ni1—O4, Ni1—O5 and Ni1—N1 bond lengths are 2.0172 (16), 2.114 (2) and 2.145 (2) Å, respectively (Table 1 ▸). The C—O bond lengths in the deprotonated carb­oxy­lic groups differ noticeably [C1—O1 = 1.250 (3) Å and C4—O4 = 1.263 (3) Å], which is typical for monodentately coordinated carboxyl­ates (Gumienna-Kontecka *et al.*, 2007 ▸; Pavlishchuk *et al.*, 2010 ▸; Penkova *et al.*, 2010 ▸). In the same way, the C5—O6, C7—O5 and C12—O7 bonds [1.431 (3), 1.440 (3) and 1.434 (3) Å, respectively] show single-bond character. The C10—N1 and C11—N1 bond lengths are similar [1.490 (3) and 1.497 (3) Å, respectively], while the C6—N2 and C9—N2 bonds are also not significantly different [1.500 (3) and 1.484 (4) Å, respectively]. An intra­molecular C14—H14*B*⋯O4 hydrogen bond occurs while the complex mol­ecule and water mol­ecules are linked by O—H⋯O hydrogen bonds (O9—H9*C*⋯O8, O9—H9*D*⋯O10, O10—H10*D*⋯O11, O11—H11*C*⋯O12, O11—H11*D*⋯O3; Fig. 1 ▸ and Table 2 ▸).
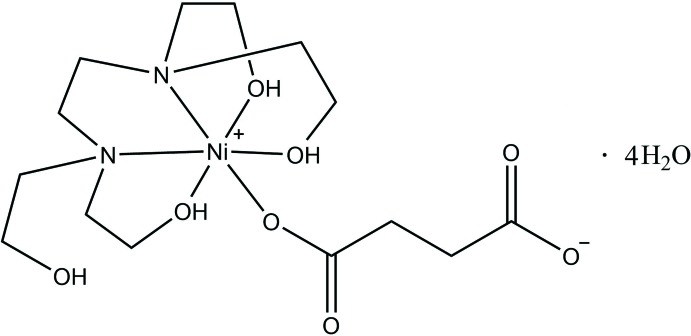


## Supra­molecular features   

**Figure 2:**
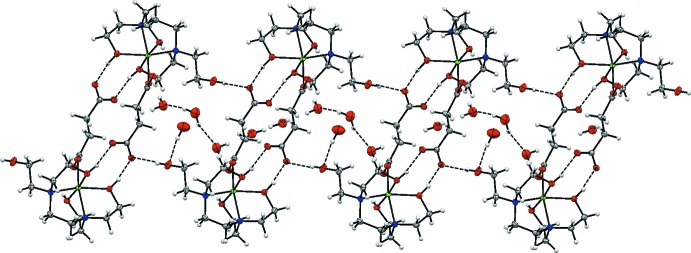
A view of the crystal packing of the title compound along the *c* axis. Dashed lines denote the intra­molecular and inter­molecular hydrogen bonds.

The crystal packing of the title compound (Fig. 2 ▸) features inter­molecular hydrogen bonds (O5—H5⋯O2^i^, O7—H7⋯O1^i^, O8—H8⋯O2^ii^, O10—H10*C*⋯O11^iii^, O12—H12*C*⋯O1^iv^ and C6—H6*A*⋯O10^v^; symmetry codes as in Table 2 ▸), which connect the mol­ecules into a three-dimensional supra­molecular architecture. All four O atoms of the water mol­ecules are involved in intra or inter­molecular hydrogen bonds.

## Database survey   

A search of the Cambridge Structural database (CSD, version 5.39, update May 2018; Groom *et al.*, 2016 ▸) revealed that there are several precendents for *catena*-{[[*N*,*N*,*N*′,*N*′-tetra­kis­(2-hy­droxy­eth­yl)ethyl­enedi­amine-κ^2^ *N* ^1^,*N* ^2^]nickel(II)]-μ-succinato-κ*O* ^4^} tetra­hydrate, including the structures of hexa­aqua­nickel(II) bis­{aqua[*N*-(2-{bis­[(carb­oxy)meth­yl]amino}­eth­yl)gly­cinato]nickel(II)} dihydrate (NELMUO; Belošević *et al.*, 2013 ▸), hexa­aqua­nickel(II) (μ^2^-tri­ethyl­ene­tetra-amine­hexa­acetato)­diaqua­dinickel(II) dihydrate (UCAWEB; Shi *et al.*, 2006 ▸) and sodium aqua­{hydrogen 2,2′,2′′,2′′′-[ethane-1,2-diylbis(nitrilo)]tetra­acetato}­nickel(II) trihydrate (WAPHAY; Crouse *et al.*, 2012 ▸). In addition, tetra­aqua­bis­(isonicotinamide-κ*N* ^1^)nickel(II) bis­(4-formyl­benzo­ate) dihydrate (HUCLAT; Hökelek *et al.*, 2009 ▸), *trans*-tetra­aqua­bis­(isonicotinamide)­nickel(II) bis­(3-hy­droxy­benzo­ate) tetra­hydrate (GANZAY; Zaman *et al.*, 2012 ▸) and tetra­aqua­bis­(isonicotinamide)­nickel(II) thio­phene-2,5-di­carboxylate dihydrate (NETQIO; Liu *et al.*, 2012 ▸) have been reported. In these three complexes, the Ni—N bond lengths vary from 1.999 to 2.118 Å. In the title complex, the Ni—N bond lengths [2.145 (2) and 2.069 (2) Å] fall within these limits.

## Hirshfeld surface analysis   

**Figure 3:**
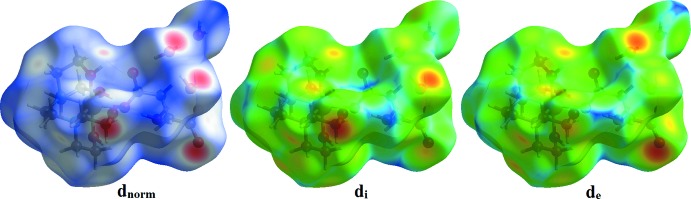
The Hirshfeld surfaces of the title compound mapped over *d*_norm_, *d*_i_ and *d*_e_.

Hirshfeld surface analysis was used to investigate the presence of hydrogen bonds and inter­molecular inter­actions in the crystal structure and two-dimensional fingerprint plots were calculated using *CrystalExplorer* (Turner *et al.*, 2017 ▸). The mol­ecular Hirshfeld surfaces were performed using a standard (high) surface resolution with the three-dimensional *d_norm_* surfaces mapped over a fixed colour scale of −0.7407 (red) to 1.6068 (blue) a.u. The red spots on the surface indicate the inter­molecular contacts involved in the hydrogen bonds. The red spots identified in Figs. 3 ▸ and 4 ▸ correspond to the near-type H⋯O contacts resulting from O—H⋯O and C—H⋯O hydrogen bonds (Table 2 ▸).

**Figure 5:**
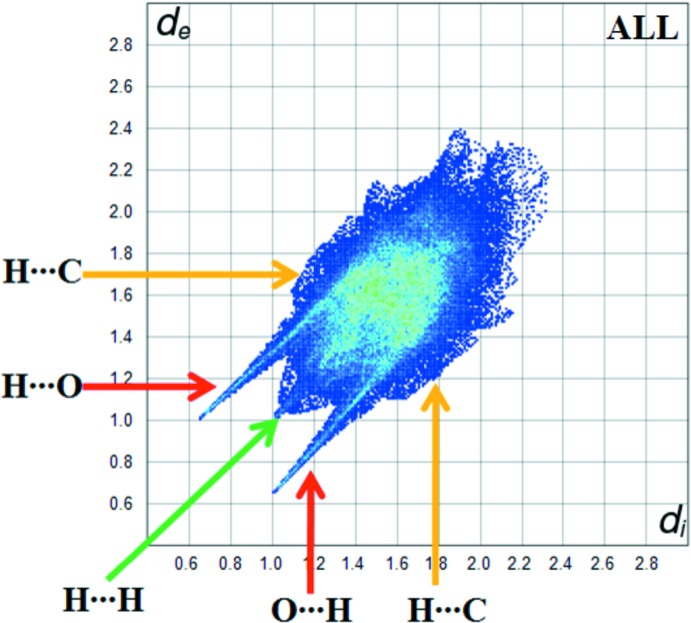
The fingerprint plot for all inter­actions.

**Figure 6:**
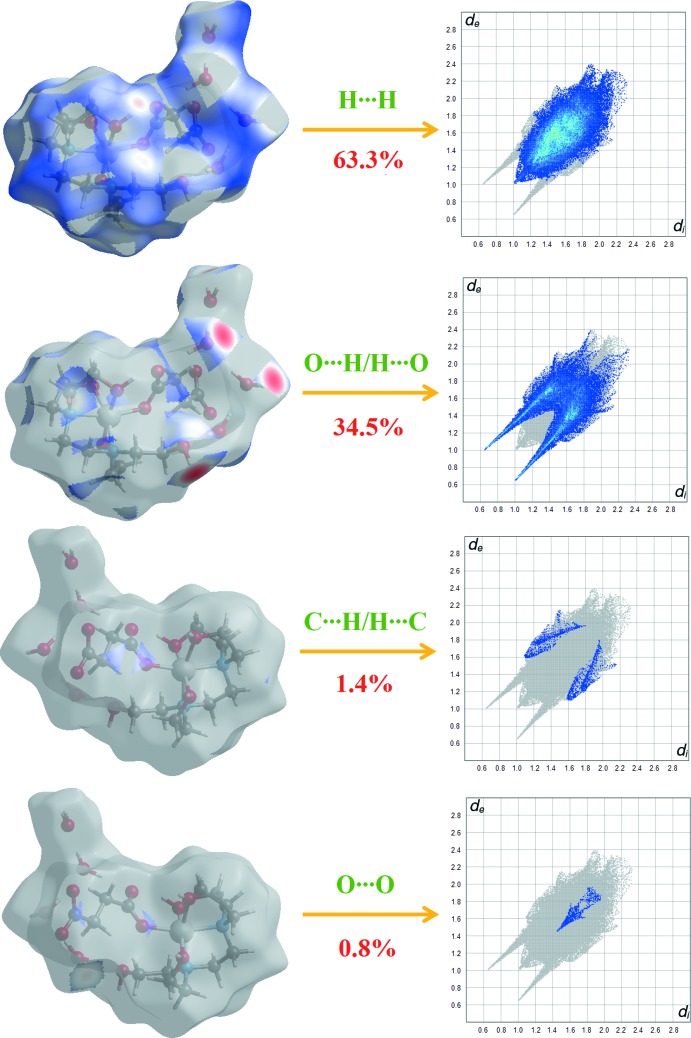
Two-dimensional fingerprint plots with a *d*_norm_ view of the H⋯H (63.3%), O⋯H/H⋯O (34.5%), C⋯H/H⋯C (1.4%) and O⋯O (0.8%) contacts in the title compound.

Fig. 5 ▸ shows the two-dimensional fingerprint plot for the sum of the contacts contributing to the Hirshfeld surface represented in normal mode. The graph shown in Fig. 6 ▸ represents the O⋯H/H⋯O contacts (34.5%) between the oxygen atoms inside the surface and the hydrogen atoms outside the surface, *d*_e_ + *d*_i_ = 1.7 Å, and has two symmetrical points at the top, bottom left and right. These data are characteristic of O—H⋯O and C—H⋯O hydrogen bonds (Table 2 ▸). The top plot shown in Fig. 6 ▸ shows the two-dimensional fingerprint of the (*d*_i_, *d*_e_) points associated with hydrogen atoms. It is characterized by an end point that points to the origin and corresponds to *d*_i_ = *d*_e_ = 1.0 Å, which indicates the presence of the H⋯H contacts (63.3% contribution). The graph for C⋯H/H⋯C represents the contacts ((1.4% contribution) between the carbon atoms inside the Hirshfeld surface and the hydrogen atoms outside it and *vice versa*. It has two symmetrical wings on the left and right sides.

**Figure 7:**
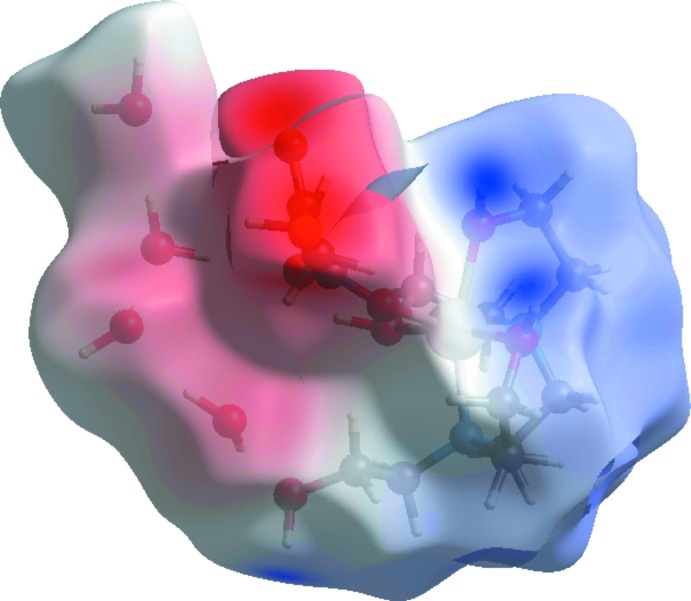
Hirshfeld surface plotted over electrostatic potential energy.

In the view of the three-dimensional Hirshfeld surface of the title compound plotted over electrostatic potential energy in the range −0.308 to 0.257 a.u. using the STO-3G basis set at the Hartree–Fock level of theory, Fig. 7 ▸, the C—H⋯O and O—H⋯O hydrogen-bond donors and acceptors are shown as blue and red areas around the atoms with positive (hydrogen-bond donors) and negative (hydrogen-bond acceptors) electrostatic potentials, respectively.

## Synthesis and crystallization   

A solution of NaOH (50 mmol, 2.0 g) was added to an aqueous solution of succinic acid (25 mmol, 3 g) under stirring. A solution of NiCl_2_·6H_2_O (25 mmol, 6.14 g) in methanol was added. The mixture was heated at 353 K for one h and then the blue mixture was filtered and left to dry at room temperature. The product (0.88 mmol, 0.20 g) was dissolved in ethanol and added to a ethanol solution of *N*,*N*,*N*′,*N*′-tetra­kis­(2-hy­droxy­eth­yl)ethyl­enedi­amine (1.75 mmol, 0.41 g). The mixture was heated at 353 K for one h under stirring and the resulting suspension was filtered. It was allowed to crystallize for four weeks at room temperature. Blue prismatic crystals suitable for X-ray diffraction analysis were obtained.

## Refinement   

Crystal data, data collection and structure refinement details are summarized in Table 3 ▸. C-bound H atoms were geometrically positioned with C—H distances of 0.93–0.97 Å. and refined as riding, with *U*_iso_(H) = 1.2*U*_eq_(C). N-bound H atoms were located in difference-Fourier maps and refined isotropically. The water H atoms were located in a difference map and were refined subject to a DFIX restraint of O—H = 0.85 Å. The O12—H12*C* bond length was refined with a DFIX restraint of 0.84 (4) Å. The H atoms bonded to other O atoms (O5, O6, O7 and O8) were located in a difference map and refined freely.

## Supplementary Material

Crystal structure: contains datablock(s) I. DOI: 10.1107/S2056989018015359/xu5943sup1.cif

Structure factors: contains datablock(s) I. DOI: 10.1107/S2056989018015359/xu5943Isup2.hkl

CCDC reference: 1564209

Additional supporting information:  crystallographic information; 3D view; checkCIF report

## Figures and Tables

**Figure 4:**
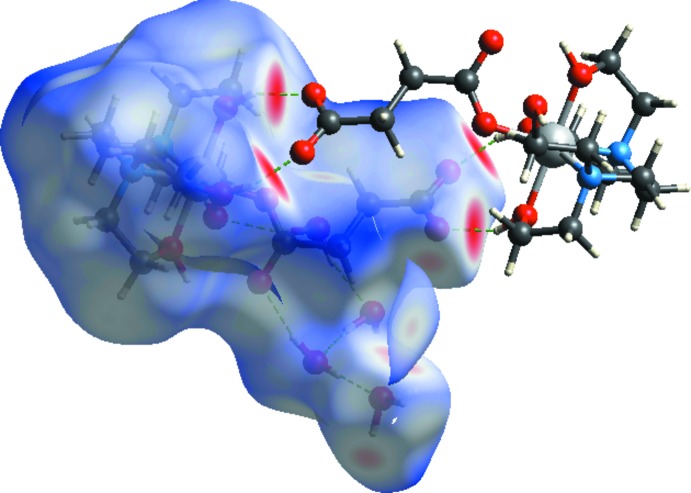
Hirshfeld surface mapped over *d*_norm_ to visualize the inter­molecular inter­actions.

**Table 1:** Selected geometric parameters (Å, °)

Ni1—O4	2.0172 (16)	Ni1—O5	2.114 (2)
Ni1—O6	2.0622 (18)	Ni1—N1	2.145 (2)
Ni1—N2	2.069 (2)	O4—C4	1.263 (3)
Ni1—O7	2.0768 (17)	O1—C1	1.250 (3)
			
O4—Ni1—N2	165.11 (9)	O4—Ni1—N1	108.35 (8)
O6—Ni1—O7	174.18 (7)	N2—Ni1—N1	85.82 (9)
O6—Ni1—O5	95.59 (8)	O5—Ni1—N1	162.10 (8)
			
Ni1—O4—C4—O3	29.5 (4)	Ni1—N1—C10—C9	36.8 (3)
Ni1—O4—C4—C3	−147.4 (2)	Ni1—O7—C12—C11	56.1 (2)

**Table 2:** Hydrogen-bond geometry (Å, °)

*D*—H⋯*A*	*D*—H	H⋯*A*	*D*⋯*A*	*D*—H⋯*A*
O5—H5⋯O2^i^	0.86	1.76	2.585 (3)	158
O6—H6⋯O3	0.87	2.03	2.581 (2)	121
O7—H7⋯O1^i^	0.87	1.80	2.603 (3)	152
O8—H8⋯O2^ii^	0.82	1.87	2.687 (3)	175
O9—H9*C*⋯O8	0.85	1.98	2.803 (4)	162
O9—H9*D*⋯O10	0.85	1.94	2.767 (6)	164
O10—H10*C*⋯O11^iii^	0.85	2.09	2.892 (5)	156
O10—H10*D*⋯O11	0.85	2.10	2.913 (5)	160
O11—H11*C*⋯O12	0.85	1.99	2.836 (4)	178
O11—H11*D*⋯O3	0.85	2.02	2.865 (4)	172
O12—H12*C*⋯O1^iv^	0.82 (4)	2.38 (5)	2.915 (4)	124 (5)
C6—H6*A*⋯O10^v^	0.97	2.57	3.458 (5)	152
C14—H14*B*⋯O4	0.97	2.39	3.313 (4)	158

Symmetry codes: (i) 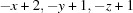; (ii) 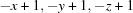; (iii) 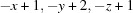; (iv) 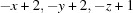; (v) 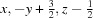.

**Table 3:** Experimental details

Crystal data	
Chemical formula	[Ni(C_10_H_24_N_2_O_4_)(C_4_H_4_O_4_)]·4H_2_O
*M*_r_	483.16
Crystal system, space group	Monoclinic, *P*2_1_/*c*
Temperature (K)	296
*a*, *b*, *c* (Å)	10.1369 (6), 10.8182 (5), 19.7771 (12)
β (°)	90.172 (5)
*V* (Å^3^)	2168.8 (2)
*Z*	4
Radiation type	Mo *K*α
μ (mm^−1^)	0.96
Crystal size (mm)	0.64 × 0.53 × 0.42

Data collection	
Diffractometer	Stoe IPDS 2
Absorption correction	Integration (*X-RED32*; Stoe & Cie, 2002 ▸)
*T*_min_, *T*_max_	0.605, 0.735
No. of measured, independent and observed [*I* > 2σ(*I*)] reflections	11333, 4472, 3581
*R*_int_	0.050
(sin θ/λ)_max_ (Å^−1^)	0.628

Refinement	
*R*[*F* ^2^ > 2σ(*F* ^2^)], *wR*(*F* ^2^), *S*	0.041, 0.113, 1.06
No. of reflections	4472
No. of parameters	283
No. of restraints	14
H-atom treatment	H atoms treated by a mixture of independent and constrained refinement
Δρ_max_, Δρ_min_ (e Å^−3^)	0.53, −0.43

Computer programs: *X-AREA* and *X-RED* (Stoe & Cie, 2002 ▸), *SHELXT2014* (Sheldrick, 2015*a* ▸), *SHELXL2018* (Sheldrick, 2015*b* ▸), *ORTEP-3 for Windows* and *WinGX* (Farrugia, 2012 ▸) and *PLATON* (Spek, 2009 ▸).

## supplementary crystallographic information

### Crystal data

**Table:**

[Ni(C_10_H_24_N_2_O_4_)(C_4_H_4_O_4_)]·4H_2_O	*F*(000) = 1032
*M**_r_* = 483.16	*D*_x_ = 1.480 Mg m^−^^3^
Monoclinic, *P*2_1_/*c*	Mo *K*α radiation, λ = 0.71073 Å
*a* = 10.1369 (6) Å	Cell parameters from 18670 reflections
*b* = 10.8182 (5) Å	θ = 1.9–27.7°
*c* = 19.7771 (12) Å	µ = 0.96 mm^−^^1^
β = 90.172 (5)°	*T* = 296 K
*V* = 2168.8 (2) Å^3^	Prism, blue
*Z* = 4	0.64 × 0.53 × 0.42 mm

### Data collection

**Table:**

Stoe IPDS 2 diffractometer	4472 independent reflections
Radiation source: sealed X-ray tube, 12 x 0.4 mm long-fine focus	3581 reflections with *I* > 2σ(*I*)
Detector resolution: 6.67 pixels mm^-1^	*R*_int_ = 0.050
rotation method scans	θ_max_ = 26.5°, θ_min_ = 2.1°
Absorption correction: integration (X-RED32; Stoe & Cie, 2002)	*h* = −11→12
*T*_min_ = 0.605, *T*_max_ = 0.735	*k* = −13→13
11333 measured reflections	*l* = −24→24

### Refinement

**Table:**

Refinement on *F*^2^	14 restraints
Least-squares matrix: full	Hydrogen site location: mixed
*R*[*F*^2^ > 2σ(*F*^2^)] = 0.041	H atoms treated by a mixture of independent and constrained refinement
*wR*(*F*^2^) = 0.113	*w* = 1/[σ^2^(*F*_o_^2^) + (0.0674*P*)^2^ + 0.0617*P*] where *P* = (*F*_o_^2^ + 2*F*_c_^2^)/3
*S* = 1.06	(Δ/σ)_max_ = 0.003
4472 reflections	Δρ_max_ = 0.53 e Å^−^^3^
283 parameters	Δρ_min_ = −0.43 e Å^−^^3^

### Special details

**Table:**

Geometry. All esds (except the esd in the dihedral angle between two l.s. planes) are estimated using the full covariance matrix. The cell esds are taken into account individually in the estimation of esds in distances, angles and torsion angles; correlations between esds in cell parameters are only used when they are defined by crystal symmetry. An approximate (isotropic) treatment of cell esds is used for estimating esds involving l.s. planes.

### Fractional atomic coordinates and isotropic or equivalent isotropic displacement parameters (Å^2^)

**Table:**

	*x*	*y*	*z*	*U*_iso_*/*U*_eq_	
Ni1	0.78305 (3)	0.48644 (3)	0.27834 (2)	0.03216 (11)	
O6	0.71228 (19)	0.64974 (17)	0.23851 (8)	0.0399 (4)	
O7	0.83482 (18)	0.31821 (16)	0.32148 (8)	0.0389 (4)	
O4	0.7758 (2)	0.55541 (16)	0.37302 (8)	0.0402 (4)	
O5	0.98373 (19)	0.53962 (19)	0.27746 (9)	0.0441 (4)	
O1	1.0038 (2)	0.6832 (2)	0.57591 (9)	0.0506 (5)	
O3	0.7525 (2)	0.75762 (18)	0.35312 (9)	0.0492 (5)	
O2	0.8782 (2)	0.5373 (2)	0.62081 (10)	0.0505 (5)	
O8	0.3602 (2)	0.5662 (2)	0.36159 (13)	0.0616 (6)	
H8	0.289310	0.530698	0.365865	0.092*	
N1	0.6030 (2)	0.3883 (2)	0.25904 (10)	0.0386 (5)	
N2	0.8286 (2)	0.4497 (2)	0.17844 (10)	0.0399 (5)	
O11	0.6745 (3)	0.9685 (3)	0.43095 (17)	0.0797 (8)	
H11C	0.743874	1.002983	0.446171	0.120*	
H11D	0.695813	0.910368	0.404223	0.120*	
C1	0.9109 (3)	0.6073 (2)	0.57295 (12)	0.0406 (6)	
C4	0.7866 (3)	0.6676 (2)	0.38981 (12)	0.0394 (6)	
O12	0.9026 (3)	1.0865 (3)	0.48350 (16)	0.0815 (8)	
C11	0.6166 (3)	0.2652 (2)	0.29282 (14)	0.0443 (6)	
H11A	0.644685	0.204241	0.259896	0.053*	
H11B	0.531595	0.239664	0.310360	0.053*	
C10	0.6021 (3)	0.3719 (3)	0.18425 (14)	0.0497 (7)	
H10A	0.566366	0.445588	0.163106	0.060*	
H10B	0.545229	0.302940	0.172542	0.060*	
C12	0.7148 (3)	0.2705 (2)	0.34949 (14)	0.0440 (6)	
H12A	0.683244	0.324126	0.385255	0.053*	
H12B	0.729239	0.188620	0.368117	0.053*	
C6	0.8042 (3)	0.5651 (3)	0.13822 (13)	0.0471 (7)	
H6A	0.722563	0.556087	0.113029	0.057*	
H6B	0.875128	0.575787	0.105906	0.057*	
C5	0.7955 (3)	0.6798 (3)	0.18258 (13)	0.0466 (6)	
H5A	0.882565	0.703352	0.198508	0.056*	
H5B	0.758527	0.748288	0.157150	0.056*	
C9	0.7392 (3)	0.3480 (3)	0.15738 (14)	0.0503 (7)	
H9A	0.771937	0.269966	0.174776	0.060*	
H9B	0.736540	0.342881	0.108427	0.060*	
O10	0.5320 (4)	0.8538 (4)	0.54221 (19)	0.1024 (10)	
H10C	0.461361	0.887695	0.555905	0.154*	
H10D	0.555241	0.886225	0.504954	0.154*	
C8	0.9674 (3)	0.4115 (3)	0.17774 (13)	0.0481 (7)	
H8A	0.998072	0.406052	0.131423	0.058*	
H8B	0.975995	0.330382	0.198239	0.058*	
C13	0.4778 (3)	0.4508 (3)	0.27750 (14)	0.0471 (6)	
H13A	0.404622	0.399473	0.262823	0.057*	
H13B	0.472178	0.528317	0.253025	0.057*	
C3	0.8508 (4)	0.6932 (3)	0.45697 (14)	0.0549 (8)	
H3A	0.944899	0.702764	0.450027	0.066*	
H3B	0.817365	0.771168	0.474064	0.066*	
C14	0.4626 (3)	0.4768 (3)	0.35170 (17)	0.0541 (7)	
H14A	0.440785	0.401067	0.375403	0.065*	
H14B	0.545096	0.508051	0.369923	0.065*	
C2	0.8295 (3)	0.5965 (3)	0.50927 (13)	0.0526 (7)	
H2A	0.847406	0.516578	0.489003	0.063*	
H2B	0.737092	0.597494	0.521824	0.063*	
C7	1.0510 (3)	0.5040 (3)	0.21648 (15)	0.0497 (7)	
H7A	1.135643	0.467410	0.227709	0.060*	
H7B	1.066719	0.576359	0.188659	0.060*	
O9	0.4560 (5)	0.6270 (4)	0.49044 (17)	0.1286 (14)	
H6	0.691139	0.719062	0.257744	0.193*	
H7	0.897531	0.295947	0.348879	0.193*	
H5	1.041734	0.531317	0.309305	0.193*	
H9C	0.418851	0.624575	0.451870	0.193*	
H9D	0.466146	0.701837	0.502467	0.193*	
H12C	0.872 (6)	1.154 (3)	0.474 (3)	0.16 (3)*	
H12D	0.980 (4)	1.105 (5)	0.497 (5)	0.31 (7)*	

### Atomic displacement parameters (Å^2^)

**Table:**

	*U*^11^	*U*^22^	*U*^33^	*U*^12^	*U*^13^	*U*^23^
Ni1	0.03494 (19)	0.03014 (17)	0.03141 (16)	−0.00050 (13)	0.00031 (11)	0.00160 (11)
O6	0.0440 (11)	0.0373 (9)	0.0383 (8)	0.0033 (8)	0.0026 (8)	0.0053 (7)
O7	0.0389 (10)	0.0347 (9)	0.0430 (9)	−0.0009 (8)	−0.0022 (7)	0.0043 (7)
O4	0.0511 (12)	0.0348 (9)	0.0347 (8)	−0.0036 (8)	0.0008 (8)	−0.0006 (7)
O5	0.0371 (10)	0.0524 (11)	0.0426 (9)	−0.0040 (9)	0.0011 (8)	0.0039 (8)
O1	0.0493 (12)	0.0560 (12)	0.0465 (10)	−0.0098 (10)	−0.0097 (9)	0.0051 (9)
O3	0.0676 (14)	0.0370 (10)	0.0430 (10)	0.0029 (10)	−0.0090 (9)	0.0004 (7)
O2	0.0459 (12)	0.0616 (12)	0.0440 (10)	−0.0027 (10)	−0.0038 (9)	0.0104 (9)
O8	0.0498 (13)	0.0501 (12)	0.0850 (15)	0.0042 (11)	0.0207 (12)	0.0023 (11)
N1	0.0355 (12)	0.0367 (11)	0.0436 (11)	−0.0007 (9)	−0.0011 (9)	0.0018 (9)
N2	0.0431 (13)	0.0413 (12)	0.0353 (10)	0.0015 (10)	0.0025 (9)	0.0001 (8)
O11	0.078 (2)	0.0564 (15)	0.105 (2)	0.0042 (14)	−0.0075 (16)	−0.0150 (14)
C1	0.0422 (15)	0.0420 (13)	0.0377 (12)	0.0054 (12)	−0.0007 (11)	−0.0029 (10)
C4	0.0412 (15)	0.0406 (13)	0.0365 (12)	−0.0024 (12)	0.0000 (10)	−0.0012 (10)
O12	0.084 (2)	0.0730 (19)	0.0877 (19)	−0.0127 (17)	−0.0047 (16)	0.0158 (15)
C11	0.0403 (15)	0.0357 (13)	0.0569 (15)	−0.0048 (12)	−0.0007 (12)	0.0024 (11)
C10	0.0470 (17)	0.0567 (17)	0.0454 (14)	−0.0053 (14)	−0.0102 (12)	−0.0041 (12)
C12	0.0466 (17)	0.0352 (13)	0.0502 (14)	−0.0007 (12)	0.0006 (12)	0.0089 (10)
C6	0.0526 (18)	0.0538 (16)	0.0349 (12)	0.0035 (14)	0.0035 (12)	0.0078 (11)
C5	0.0525 (17)	0.0430 (14)	0.0443 (13)	0.0039 (13)	0.0047 (12)	0.0128 (11)
C9	0.0567 (19)	0.0506 (16)	0.0435 (14)	−0.0023 (14)	−0.0016 (13)	−0.0111 (12)
O10	0.092 (3)	0.099 (3)	0.116 (3)	−0.004 (2)	0.0061 (19)	−0.003 (2)
C8	0.0487 (17)	0.0528 (16)	0.0428 (13)	0.0102 (14)	0.0098 (12)	0.0016 (12)
C13	0.0362 (15)	0.0455 (15)	0.0595 (16)	0.0018 (12)	−0.0024 (12)	0.0058 (12)
C3	0.078 (2)	0.0388 (14)	0.0481 (15)	−0.0040 (15)	−0.0195 (15)	−0.0021 (11)
C14	0.0411 (16)	0.0563 (17)	0.0649 (18)	0.0046 (14)	0.0074 (14)	0.0001 (14)
C2	0.0541 (19)	0.0646 (19)	0.0392 (13)	−0.0101 (15)	−0.0058 (12)	0.0018 (12)
C7	0.0404 (15)	0.0591 (18)	0.0498 (15)	0.0021 (14)	0.0105 (12)	0.0077 (13)
O9	0.180 (4)	0.106 (3)	0.100 (2)	−0.014 (3)	−0.013 (2)	−0.017 (2)

### Geometric parameters (Å, º)

**Table:**

Ni1—O4	2.0172 (16)	C11—H11B	0.9700
Ni1—O6	2.0622 (18)	C10—C9	1.512 (4)
Ni1—N2	2.069 (2)	C10—H10A	0.9700
Ni1—O7	2.0768 (17)	C10—H10B	0.9700
Ni1—O5	2.114 (2)	C12—H12A	0.9700
Ni1—N1	2.145 (2)	C12—H12B	0.9700
O6—C5	1.431 (3)	C6—C5	1.522 (4)
O6—H6	0.8680	C6—H6A	0.9700
O7—C12	1.434 (3)	C6—H6B	0.9700
O7—H7	0.8681	C5—H5A	0.9700
O4—C4	1.263 (3)	C5—H5B	0.9700
O5—C7	1.440 (3)	C9—H9A	0.9700
O5—H5	0.8650	C9—H9B	0.9700
O1—C1	1.250 (3)	O10—H10C	0.8500
O3—C4	1.262 (3)	O10—H10D	0.8501
O2—C1	1.257 (3)	C8—C7	1.518 (4)
O8—C14	1.433 (4)	C8—H8A	0.9700
O8—H8	0.8200	C8—H8B	0.9700
N1—C13	1.485 (4)	C13—C14	1.502 (4)
N1—C10	1.490 (3)	C13—H13A	0.9700
N1—C11	1.497 (3)	C13—H13B	0.9700
N2—C8	1.467 (4)	C3—C2	1.488 (4)
N2—C9	1.484 (4)	C3—H3A	0.9700
N2—C6	1.500 (3)	C3—H3B	0.9700
O11—H11C	0.8500	C14—H14A	0.9700
O11—H11D	0.8500	C14—H14B	0.9700
C1—C2	1.508 (4)	C2—H2A	0.9700
C4—C3	1.503 (4)	C2—H2B	0.9700
O12—H12C	0.816 (10)	C7—H7A	0.9700
O12—H12D	0.851 (9)	C7—H7B	0.9700
C11—C12	1.498 (4)	O9—H9C	0.8500
C11—H11A	0.9700	O9—H9D	0.8500
			
O4—Ni1—O6	91.38 (7)	C11—C12—H12A	110.4
O4—Ni1—N2	165.11 (9)	O7—C12—H12B	110.4
O6—Ni1—N2	83.00 (8)	C11—C12—H12B	110.4
O4—Ni1—O7	87.29 (7)	H12A—C12—H12B	108.6
O6—Ni1—O7	174.18 (7)	N2—C6—C5	112.5 (2)
N2—Ni1—O7	99.62 (8)	N2—C6—H6A	109.1
O4—Ni1—O5	86.84 (8)	C5—C6—H6A	109.1
O6—Ni1—O5	95.59 (8)	N2—C6—H6B	109.1
N2—Ni1—O5	80.03 (8)	C5—C6—H6B	109.1
O7—Ni1—O5	90.00 (7)	H6A—C6—H6B	107.8
O4—Ni1—N1	108.35 (8)	O6—C5—C6	107.2 (2)
O6—Ni1—N1	93.50 (8)	O6—C5—H5A	110.3
N2—Ni1—N1	85.82 (9)	C6—C5—H5A	110.3
O7—Ni1—N1	81.56 (8)	O6—C5—H5B	110.3
O5—Ni1—N1	162.10 (8)	C6—C5—H5B	110.3
C5—O6—Ni1	106.55 (15)	H5A—C5—H5B	108.5
C5—O6—H6	106.8	N2—C9—C10	109.6 (2)
Ni1—O6—H6	131.2	N2—C9—H9A	109.7
C12—O7—Ni1	105.11 (15)	C10—C9—H9A	109.7
C12—O7—H7	106.2	N2—C9—H9B	109.7
Ni1—O7—H7	133.1	C10—C9—H9B	109.7
C4—O4—Ni1	126.59 (16)	H9A—C9—H9B	108.2
C7—O5—Ni1	113.10 (17)	H10C—O10—H10D	109.5
C7—O5—H5	105.0	N2—C8—C7	110.1 (2)
Ni1—O5—H5	128.2	N2—C8—H8A	109.6
C14—O8—H8	109.5	C7—C8—H8A	109.6
C13—N1—C10	107.2 (2)	N2—C8—H8B	109.6
C13—N1—C11	111.9 (2)	C7—C8—H8B	109.6
C10—N1—C11	109.7 (2)	H8A—C8—H8B	108.2
C13—N1—Ni1	117.32 (17)	N1—C13—C14	114.5 (2)
C10—N1—Ni1	103.81 (17)	N1—C13—H13A	108.6
C11—N1—Ni1	106.49 (16)	C14—C13—H13A	108.6
C8—N2—C9	111.9 (2)	N1—C13—H13B	108.6
C8—N2—C6	112.7 (2)	C14—C13—H13B	108.6
C9—N2—C6	111.6 (2)	H13A—C13—H13B	107.6
C8—N2—Ni1	106.26 (15)	C2—C3—C4	114.9 (2)
C9—N2—Ni1	105.82 (16)	C2—C3—H3A	108.5
C6—N2—Ni1	108.03 (16)	C4—C3—H3A	108.5
H11C—O11—H11D	109.5	C2—C3—H3B	108.5
O1—C1—O2	124.1 (2)	C4—C3—H3B	108.5
O1—C1—C2	120.0 (2)	H3A—C3—H3B	107.5
O2—C1—C2	116.0 (3)	O8—C14—C13	109.6 (3)
O3—C4—O4	124.6 (2)	O8—C14—H14A	109.7
O3—C4—C3	118.8 (2)	C13—C14—H14A	109.7
O4—C4—C3	116.5 (2)	O8—C14—H14B	109.7
H12C—O12—H12D	101.8 (15)	C13—C14—H14B	109.7
N1—C11—C12	111.1 (2)	H14A—C14—H14B	108.2
N1—C11—H11A	109.4	C3—C2—C1	116.5 (3)
C12—C11—H11A	109.4	C3—C2—H2A	108.2
N1—C11—H11B	109.4	C1—C2—H2A	108.2
C12—C11—H11B	109.4	C3—C2—H2B	108.2
H11A—C11—H11B	108.0	C1—C2—H2B	108.2
N1—C10—C9	111.5 (2)	H2A—C2—H2B	107.3
N1—C10—H10A	109.3	O5—C7—C8	109.5 (2)
C9—C10—H10A	109.3	O5—C7—H7A	109.8
N1—C10—H10B	109.3	C8—C7—H7A	109.8
C9—C10—H10B	109.3	O5—C7—H7B	109.8
H10A—C10—H10B	108.0	C8—C7—H7B	109.8
O7—C12—C11	106.7 (2)	H7A—C7—H7B	108.2
O7—C12—H12A	110.4	H9C—O9—H9D	109.5
			
Ni1—O4—C4—O3	29.5 (4)	Ni1—N2—C9—C10	42.5 (3)
Ni1—O4—C4—C3	−147.4 (2)	N1—C10—C9—N2	−55.8 (3)
C13—N1—C11—C12	−105.5 (3)	C9—N2—C8—C7	−165.1 (2)
C10—N1—C11—C12	135.7 (3)	C6—N2—C8—C7	68.1 (3)
Ni1—N1—C11—C12	23.9 (3)	Ni1—N2—C8—C7	−50.0 (2)
C13—N1—C10—C9	161.6 (2)	C10—N1—C13—C14	−179.1 (3)
C11—N1—C10—C9	−76.7 (3)	C11—N1—C13—C14	60.6 (3)
Ni1—N1—C10—C9	36.8 (3)	Ni1—N1—C13—C14	−62.9 (3)
Ni1—O7—C12—C11	56.1 (2)	O3—C4—C3—C2	151.7 (3)
N1—C11—C12—O7	−54.5 (3)	O4—C4—C3—C2	−31.2 (4)
C8—N2—C6—C5	−99.8 (3)	N1—C13—C14—O8	163.5 (2)
C9—N2—C6—C5	133.2 (3)	C4—C3—C2—C1	169.7 (3)
Ni1—N2—C6—C5	17.3 (3)	O1—C1—C2—C3	−10.1 (4)
Ni1—O6—C5—C6	50.5 (2)	O2—C1—C2—C3	169.4 (3)
N2—C6—C5—O6	−45.8 (3)	Ni1—O5—C7—C8	−13.7 (3)
C8—N2—C9—C10	157.8 (2)	N2—C8—C7—O5	42.3 (3)
C6—N2—C9—C10	−74.8 (3)		

### Hydrogen-bond geometry (Å, º)

**Table:**

*D*—H···*A*	*D*—H	H···*A*	*D*···*A*	*D*—H···*A*
O5—H5···O2^i^	0.86	1.76	2.585 (3)	158
O6—H6···O3	0.87	2.03	2.581 (2)	121
O7—H7···O1^i^	0.87	1.80	2.603 (3)	152
O8—H8···O2^ii^	0.82	1.87	2.687 (3)	175
O9—H9*C*···O8	0.85	1.98	2.803 (4)	162
O9—H9*D*···O10	0.85	1.94	2.767 (6)	164
O10—H10*C*···O11^iii^	0.85	2.09	2.892 (5)	156
O10—H10*D*···O11	0.85	2.10	2.913 (5)	160
O11—H11*C*···O12	0.85	1.99	2.836 (4)	178
O11—H11*D*···O3	0.85	2.02	2.865 (4)	172
O12—H12*C*···O1^iv^	0.82 (4)	2.38 (5)	2.915 (4)	124 (5)
C6—H6*A*···O10^v^	0.97	2.57	3.458 (5)	152
C14—H14*B*···O4	0.97	2.39	3.313 (4)	158

Symmetry codes: (i) −*x*+2, −*y*+1, −*z*+1; (ii) −*x*+1, −*y*+1, −*z*+1; (iii) −*x*+1, −*y*+2, −*z*+1; (iv) −*x*+2, −*y*+2, −*z*+1; (v) *x*, −*y*+3/2, *z*−1/2.
